# Ethnobotany of Indigenous Saraguros: Medicinal Plants Used by Community Healers “Hampiyachakkuna” in the San Lucas Parish, Southern Ecuador

**DOI:** 10.1155/2017/9343724

**Published:** 2017-07-04

**Authors:** José M. Andrade, Hernán Lucero Mosquera, Chabaco Armijos

**Affiliations:** ^1^Department of Chemistry, Universidad Técnica Particular de Loja, P.O. Box 11-01-608, Loja, Loja Province, Ecuador; ^2^Department of Natural Sciences, Universidad Técnica Particular de Loja, P.O. Box 11-01-608, Loja, Loja Province, Ecuador

## Abstract

This paper reports the results of an ethnobotanical survey on the use of medicinal plants by community healers “Hampiyachakkuna” in the San Lucas Parish, province of Loja, Ecuador. A particular ethnic group, the indigenous Saraguros, inhabits this region. This study reports 183 plant species used in 75 different curative therapies by the Saraguro healers.

## 1. Introduction

The Saraguros are one of the Kichwa indigenous communities of Ecuador. Although their origin is uncertain, they are considered to have been forced by the Incas to reach Ecuador from far away. A commonly accepted theory on how they reached south Ecuador is as a result of an “ethnical mobilization,” a common practice established by the great Inca Tupac Yupanqui. This strategy was used in order to secure the peace inside the Empire. As such, and according to Uhle [1], a small group of Paltas, the autochthonous inhabitants of the Loja region in south Ecuador, were transferred to Bolivia; and at the same time, a certain number of inhabitants of the Bolivian Highland Plateau were relocated in south Ecuador [2]. Nowadays, the Saraguros are normally settled in communitarian land in the southern Ecuador provinces of Loja and Zamora Chinchipe. In the canton of Loja, the Saraguros live in the San Lucas Parish, where this research was conducted.

The Saraguros are one of the best-organized ethnical groups in Ecuador and have conserved many aspects of their ancient culture and traditions for centuries. They demonstrate the latter by preserving their typical dressing, language, religion, gastronomy, architecture, social habits, and medical practices [3]. Among their medical practice traditions, this ethnic group is known for the use of medicinal plants in their own health care system. In fact, the use of these plants as therapeutic agents is an important feature of traditional indigenous medicine and is still practiced within the Saraguro community [4]. In particular, the Saraguros are highly recognized for the development of optimization techniques that help them select natural/plant resources to be used in their health care practices [5].

The community of healers locally known as “Hampiyachakkuna” maintains the ancient medical treatments of the Saraguros. The “Yachak” or “Hampi yachakkuna” is the person who knows the curative properties of plants, animals, and/or minerals. Under the Andean cosmovision of the Saraguros ethnical group, the diseases they treat are thought to be produced by either cold or heat [6]. As such, their natural medicines are classified as hot and fresh [7]; and depending on the nature of the patient's condition, different plants are selected for the treatment in accordance with this classification. However, although the knowledge regarding the usage of plants for medicinal practices has been transmitted orally from generation to generation [8], the Saraguros are experimenting cultural changes that threaten the preservation of their ancestral knowledge. These cultural changes lead to negative consequences such as the loss of traditional knowledge, a decline in the use of natural resources, and changes in the patterns of food intake, medical treatment, and, furthermore, their cosmovision. For these reasons, there is an urgent need to document and preserve their invaluable knowledge [9].

In this sense, a number of ethnobotanical studies have been conducted in Ecuador. [10–16]. More recently, a series of important contributions to ethnobotanical research in the South of Ecuador have been published [17, 18]. For example, there are studies related to the use of wild fruits as alimentary supplements [19], the documentation of the herbaceous plants of Vilcabamba [20], and of medicinal plants used in the province of Loja [21]. In the latter, the existence of more than 200 medicinal plants was reported. In the Saraguro region, only few ethnobotanical studies have reported the usefulness of different natural plants in a variety of applications [22–24]. However, to date, a thorough documentation of the plants used as medicinal resources by the healers of the Saraguro ethnical group, which is the motivation for this investigation, has not been reported.

Because of the increasing recognition of the importance of the different medicinal species used by the Saraguros and in an effort to preserve their knowledge, in this work we seek to contribute to the conservation strategy on the sustainable uses of the Ecuadorian medicinal biodiversity. The latter is considered a fundamental step in order to raise awareness of its cultural value and the importance of its preservation. By doing that, we intended to safeguard the popular knowledge concerning natural medicinal plants and to provide a baseline for future actions regarding scientific research programs, environmental education, social awareness, and sustainable natural resources exploitation. As such, this study was conducted under a technical and scientific cooperation among the Universidad Técnica Particular de Loja (UTPL), the Dirección Provincial de Salud de Loja (DPSL), and the Saraguros Healers Council (Consejo de Sanadores de Saraguro) with the objective of recognizing and recovering the traditional knowledge of herbal medicinal resources used by the Saraguro community. The results of this research also aim at becoming a starting point to attract the attention of national and international tourists, in order to promote a self-sustaining development of the Saraguro community.

## 2. Methodology

**Figure 1:**
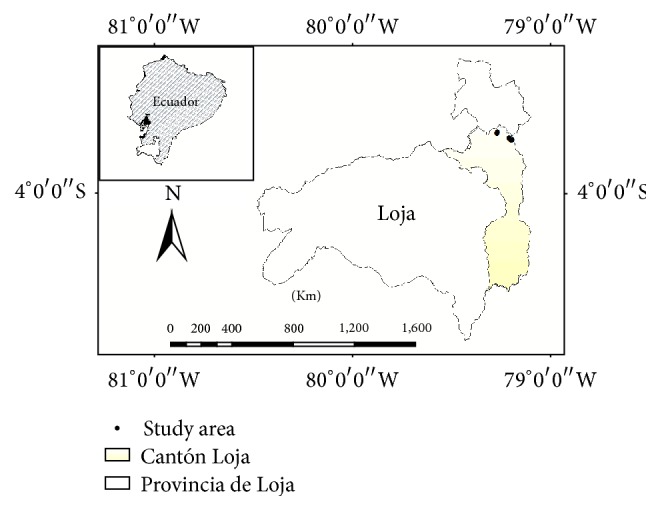
Geographical location of the San Lucas Parish, indicating the places of collection of medicinal species.

This study was carried out in the San Lucas Parish in the south Ecuadorian province of Loja (Figure 1). San Lucas is limited to the north with the Saraguro Canton and the “Loma de Oro” mountain, to the south with the Jimbilla parish by the Bunque and Puruzhuma Rivers, to the east with the Loja-Zamora Chinchipe provincial limits and the Imbana Mountain, and to the west with the Santiago parish. San Lucas has an area of 15.900 ha and a population of approximately 4,296 inhabitants [12]. The dominant ecosystem in the zone is classified as lower montane-humid forest (hf-LM) according to Holdrige classification system. It is located at an average elevation of 2,525 m a.s.l and has an irregular topography. The study area has a temperate climate, with temperature ranging between 12 and 18°C along the year [12]. Annual precipitation amounts range between 600 and 1,000 mm yr^−1^. The rainfall regime is semihumid with low seasonality.

**Figure 2:**
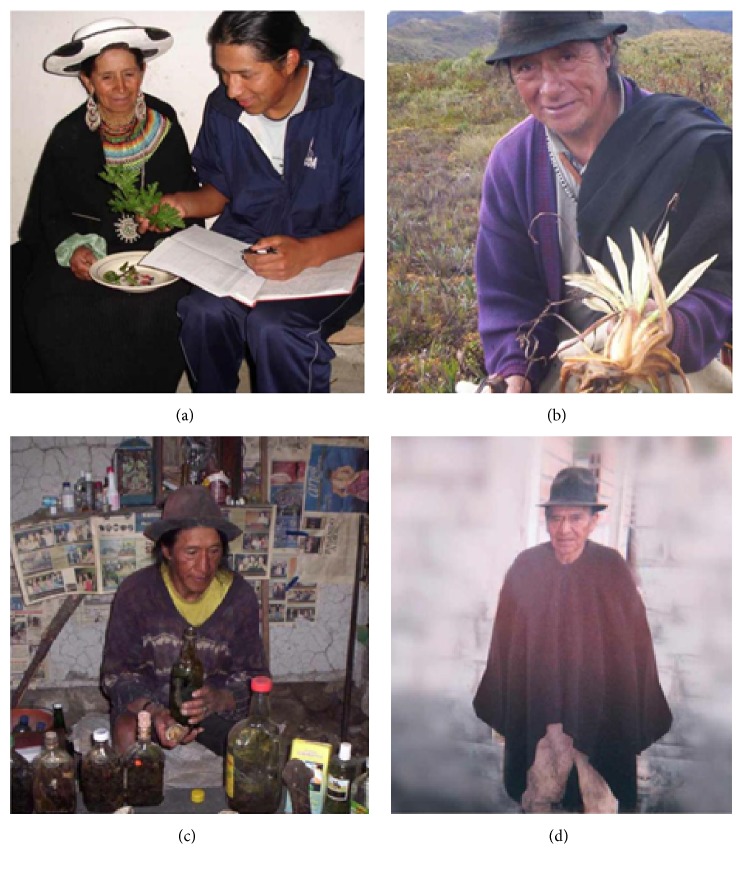
Community healers from San Lucas Parish: (a) midwife “Wachakhampiyachak” during an interview with one of the coauthors; (b) herbalist “Yurakhampiyachak”; (c) visionary “Rikuhampiyachak”; and (d) bone-healer “Kakuyhampiyachak.”

This research was carried out during 11 field trips conducted during the period June–September 2010. During these field visits, interviews with four key informants (i.e., healers) from the Saraguro community regarding the medicinal plants they use in their practices and their applications were carried out. These healers were a midwife “Wachakhampiyachak,” a herbalist “Yurakhampiyachak,” a bone-healer “Kakuyhampiyachak,” and a visionary “Rikuyhampiyachak.” All of them are inhabitants of San Lucas community (Table 1) (Figure 2).

In the Saraguro community, the midwifes (locally known as “parteras” or wachak in Kichwa) watch over the health of women in labor (“parturienta” in Spanish), prior, during, and after the child's birth, as well as during the first years of the newborn's life. They are mainly recognized for using medicinal plants normally grown in her own orchard, which facilitates their work. The herbalists (locally known as “hierbateros”) treat diseases with symptomatology of organic type. These include headache, stomach ache, articulation pain, fever, and/or allergies. The “hierbateros” mainly use medicinal resources collected in high-elevation mountainous areas. As such, differently from the “parteras,” they use larger amounts of herbal wild species during their practices. The bone-healers (locally known as “sobador”) is an empirical traumatologist who uses medicinal plants and the fat of different animals to treat the rupture of bones, sprains, and dislocations. The “visionario” (locally known as “Yachak”) are specialized in the diagnosis and treatment of diseases of supernatural nature (e.g., evil eye, fright (or “susto” in Spanish)), but also the aforementioned diseases of organic nature. They are particularly recognized for their expertise in the preparation of psychoactive potions using hallucinogen natural plants and depending of the Yachak expertise and knowledge the use of additional nonhallucinogen plants that act as psychoactive additives. For example, on the use of wamingas and trencillas (*Huperzia* spp.) for the preparation of the hallucinogenic cactus San Pedro* (Echinopsis pachanoi)* [11]. Although the practices of these community healers are highly trusted and recognized as effective within the Saraguro community, when they detect serious conditions in the health of a patient, they immediately transfer the patient to a health center or hospital.

The informants were contacted through authorized representatives of the Department of Indigenous Health (Departamento de Salud Indígena) of the Loja City Health Direction. The informants were between 60 and 80 years old, with at least 25 years of experience in the use of medicinal plant species. The consent of each of the interviewed Saraguro healers was obtained before starting the study and reporting the results. Their knowledge of traditional medicine was inherited from ancestors and close relatives. The information collected during the interviews with the healers was related to the different uses, application forms, preparation, method/s of collection, parts, and spread of the different plants used by each of them as medicinal resources.

In addition, the medicinal plants species used by each of the healers within the San Lucas Parish were collected. The species were classified according to the Red Book of endemic plants of Ecuador [21] and the Catalogue of the Vascular Plants of Ecuador [25]. After their classification, the collected specimens were deposited in the herbarium of the Plant of Natural Products (Planta de Productos Naturales) of the Universidad Técnica Particular de Loja for future studies. The qualitative evaluation and quantitative information collected of all species were tabulated and analyzed with their vernacular names, occurrence, growing places, therapeutically applications, parts used, plants habit development, and forms of preparation as described by each informant. The scientific nomenclature was recorded according to the Catalogue of the Vascular Plants of Ecuador [25]. This research was conducted under permission of the Ministerio del Ambiente del Ecuador (MAE-N°001-IC-FLO-DBAP-VS-DRLZCH-MA). Additionally, voucher specimens were prepared and deposited in Herbarium of the Universidad Técnica Particular de Loja. Date of plant species, vernacular name, scientific name and family, medicinal use, parts used and modality of preparation, form of administration, and the species used for each “Hampi Yachak” are reported in Tables 4to 7.

## 3. Results and Discussion

In this ethnobotanical survey, we identified 183 plants used by the community healers “Hampi yachakkuna” of the Saraguro ethnic in the San Lucas Parish. These were grouped into: 68 families, 129 genera, and 179 species. The most representative families were Asteraceae (30 species); Lamiaceae (14 species), Arecaceae (9 species), Solanaceae and Geraniaceae (8 species each), Amaryllidaceae and Brassicaceae (7 species each), and Piperaceae, Lycopodiaceae, and Ericaceae (6 species each). In relation to the treated diseases we found that 47 species are used for mythological treatments, 24 species for nervous system treatments, 13 species for cold treatments, 12 species for infection treatments, 9 species for general malaise treatments, and 8 species for inflammatory treatments of the liver and kidneys. These results are corroborated by the studies of [26–28]. As reported by [3, 5], as a result of their ancient Andean world view, supernatural and mythological diseases are the most commonly treated conditions by the Saraguro healers.

The type of species used by the healers, with exception of the midwifes (“Wachakhampiyachak”) who use a large amount of self-cultivated species, are wild species (57.4%). These species are generally collected at high-elevation in the highlands surrounding the parish. About the vegetative organ of the plants used, we found that the highest proportion corresponds to the use of the whole plant (30.1%), followed by the branches (21.9%), flowers (18.6%), leaves (16.4%), bark (2.2%), seeds (1.1%), and tubers (0.6%). These results contrast with those documented by [29] in the San Lucas Parish, who reported that the most commonly used part of the plants are the leaves. With regard to the preparation of the medicinal treatment products, crushing of the plants or their parts was determined as the most commonly applied method to process the rough plant tissues (28.4%), because it allows for a more effective treatment of the diseases according to the key informants. This method is followed by boiling the plant tissues in water (27.9%), infusions (25.7%), and their direct use (18%) as has been previously reported by [28].

The interviewees did not know the form of reproduction of the majority of the species documented in the study (55.2%). From the ones they knew, 27.3% have an asexual reproduction and 17.5% have a sexual reproduction (Table 2). Only 29% of the total number of the registered species (53 species) have not been previously reported in scientific investigations of phytochemical character and their pharmacological activity. In contrast, 71% (130 species) have registered studies of pharmacological and phytochemical nature. Regarding the administration and/or application of medicinal preparations, five procedures were identified: oral administration (110 species), topical administration (45 species), administration during water baths (17 species), administration during rituals (locally known as “limpias”) (9 species), and administration during steam baths (2 species).

**Figure 3:**
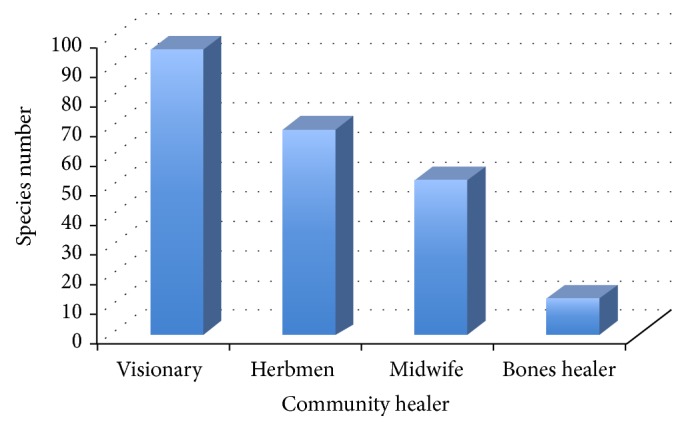
Number of species used by each community healer.

Of the total screened plants, 55.2% are native, 37.2% are introduced, and 7.7% are endemic. Similar results have been reported in the paste [20, 30]. From the 13 endemic species reported (Table 3), 2 species are used by the midwife, 3 species by the herbalist, 6 species by the visionary, and 2 species by the bone-healer. From the total number of species used by the healers, 96 are used by the visionary, 69 by the herbalist, 52 by the midwife, and 12 by the bones healer (Figure 3). It is important to mention that some of the species are used by more than one Yachak.

**Figure 4:**
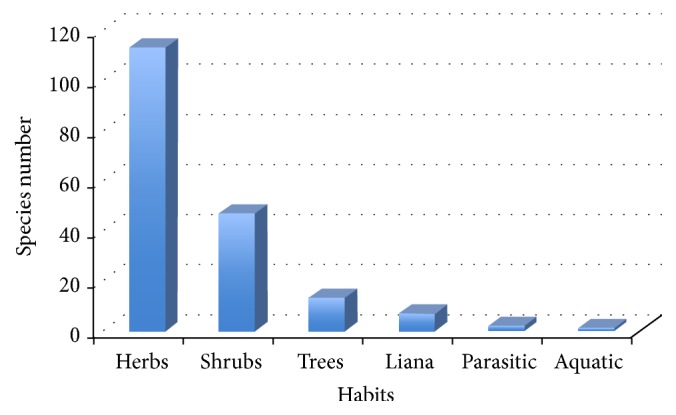
Number of species in relation to their habit.

In relation to the type of plants used by the healers, the results show that 61.8% correspond to herbs (113 species), 25.7% correspond to shrubs (47 species), 7.1% correspond to trees (13 species), 3.8% correspond to lianas (7 species), and the rest correspond to two parasitic and one aquatic species [31] (Figure 4). From these, the species grown in the “páramo” (tropical alpine grassland ecosystem) belong to* Huperzia* and* Lycopodium* genera as was previously reported by [32]. Out of the total species registered, two of them (*Bejaria resinosa* and* Huperzia*) have been studied in detail, showing a high potential of the Saraguro flora as a source of novel secondary metabolites and biologically active plants extracts as has been previously reported [33–35]. Finally, a summary of the documented plant species used by the different healers that include information of their scientific name, way of preparation, and administration is reported in Tables 4−7.

## 4. Conclusions

In this study we collected, organized, and documented the natural plants used in traditional healing practices of the Saraguro community of the San Lucas Parish in south Ecuador. We achieved this according to the directions of the World Health Organization (OMS), which is one of the prioritized strategic research lines of the National Secretariat for Science and Technology of Ecuador (SENESCYT), that is, to strengthen and enhance the recovery of ancestral knowledge in coexistence with scientific knowledge. We documented the existence of 183 species used in 75 different curative therapies by four key community healers of the Saraguro ethnic group: a midwife, an herbalist, a bone-healer, and a visionary.

This research conducted in collaboration with the members of the native Saraguro community constitutes a baseline study to help promote the preservation of this ancient medicinal knowledge by a thorough documentation of the natural resources and processing methods used. Moreover, we hope the results of this study motivate young generations to envision the potential of the use and application of traditional knowledge in medicinal practices. Finally, this scientific research and the results here reported aim at preserving and enhancing, as much as possible, a culture of the practice of natural ancient medicinal science, while preserving the environment, nature, life, culture, and sovereignty of the Saraguro people.

## Figures and Tables

**Table 1:** Places of collection of medicinal species used by community healer.

Place of collection		Community healer		
	Visionary	Herbalist	Midwife	Bone-healer
Acacana		x	x	x
Pichic		x	x	x
Ingapirca		x		
Inguera	x			
Aguarongo	x			
Plan de Duco	x			

**Table 2:** Knowledge on the propagation of species.

Propagation	Number of species	Percent (%)
Unknown	101	55.19
Sexual	32	17.49
Asexual	50	27.32

Total	183	100.00

**Table 3:** Endemic species reported.

Common name	Scientific name	Category
Pena de cerro	*Bejaria subsessilis *Benth.	Vulnerable
Suelda pequeña	*Dendrophthora fastigiata *Kuijt.	In danger
Chuquir agua	*Diplostephium oblanceolatum *S. F. Blake	Almost threated
Sacha pena	*Fuchsia hypoleuca *I. M. Johnst.	In danger
Wuaminga verde pequeño	*Huperzia austroecuadorica *B. Øllg.	Vulnerable
Shallshón	*Lepechinia paniculata *(Kunth)	Vulnerable
Pena rojo de monte	*Siphocampylus scandens *(Kunth). G. Don	Least preoccupation
Pegac chilca	*Ageratina dendroides *(Spreng) R.	Vulnerable
Sarcillo sacha	*Brachyotum scandens *(Bonpl.) Triana.	Least preoccupation
Monte de baño	*Diplostephium juniperinum *Cuatrec	In danger
Suelda grande	*Phoradendron parietarioides *Trel.	Not evaluated
Sacha algodón	*Achyrocline hallii *Hieron.	Vulnerable
Sp flor morado	*Salvia leucocephala *Kunth	Vulnerable

**Table 4:** Ethnopharmacological reports of medicinal species used by herb man “Yurakhampiyachak.”

Number	Scientific name	Herbarium voucher	Medicinal use	Preparation	Used part	Administration
1	*Cyclanthera pedata* (L.) Schrad. Cucurbitaceae	PPN-cu-004	Earache	Heat	Fruit	Topic
2	*Allium sativum* L. Liliaceae	PPN-li-001	Cough	Crushed	Garlic	Oral
3	*Medicago sativa* L. Fabaceae	PPN-fa-017	Circulatory problems in the blood system, particularly loss of sensation in the body extremities (e.g., hands, feet, and/or toes)	Liquefied	Leaves	Oral
4	*Phytolacca americana* L. Phytolaccaceae	PPN-ph-003	Dandruff	Crushed	Fruit	Topic
5	*Nasturtium officinale* R. Br. Brassicaceae	PPN-br-002	Malaise of the body and headache	Crushed	Whole	Oral
6	*Nasturtium officinale* R. Br. Brassicaceae	PPN-br-002	Pneumonia	Crushed	Leaves	Oral
7	*Ageratum conyzoides* L. Asteraceae	PPN-as-037	Gangrene and infection	Crushed	Whole plant	Oral
8	*Prunus serotina* Ehrh. Rosaceae	PPN-ro-010	Postpartum bath and bone pain.	Cooked	Leaves	Bath
9	*Cedrela montana* Moritz ex Turcz. Meliaceae	PPN-ml-004	Postpartum bath and bone pain.	Cooked	Leaves	Bath
10	*Aloysia triphylla* (L'Hér.) Britton, Verbenaceae	PPN-ve-002	Colds and colic	Infusion	Leaves	Oral
11	*Lepidium sp.* Brassicaceae	PPN-br-008	Fever or cold caused by cold air or strong winds (locally known as “mal aire”, i.e., “bad air” in Spanish)	Crushed	Whole plant	Oral
12	*Oxalis peduncularis* Kunth. Oxalidaceae	PPN-ox-002	Infection of the throat	Crushed	Whole plant	Topic:
13	*Oxalis spiralis* Ruiz & Pav. Oxalidaceae	PPN-ox-003	Infection of the throat	Crushed	Whole	Topic
14	*Dianthus caryophyllus* Caryophyllaceae	PPN-cd-001	Stomach pain	Infusion	Flowers	Oral
15	*Brassica oleracea* L. Brassicaceae	PPN-br-007	Inflammation of the liver and kidneys	Crushed	Stems	Oral
16	*Equisetum bogotense* Equisetaceae	PPN-eq-001	Inflammation of the liver	Cooked	Whole plant	Oral
17	*Oreocallis grandiflora* (Lam.) R. Br. Proteaceae	PPN-ti-001	Inflammation of the liver and kidneys	Cooked	Flowers	Oral
18	*Coriandrum sativum* L. Apiaceae	PPN-ap-010	Menstrual related abdominal pain	Infusion	Whole plant	Oral
19	*Tibouchina laxa* (Desr.) Cogn. Melastomataceae	PPN-me-003	Infection of the eyes in guinea pig (has not been applied in humans)	Crushed	Flowers	Ocular application
20	*Iresine herbstii* Hook. Amaranthaceae	PPN-am-001	Infection intestinal, injuries, liver and kidney	Crushed	Stems	Oral
21	*Epidendrum cochlidium* Lindl. Orchidaceae	PPN-or-006	Nerves	Infusion	Flowers	Oral
22	*Passiflora ligularis* Juss. Passifloraceae	PPN-pa-001	Diarrhea in children of 1 to 6 months of age	Warmed	Leaves	Topic
23	*Rumex tolimensis* Wedd. Polygonaceae	PPN-pl-005	Growing hair and dandruff control	Crushed	Stems	Topic (wash hair)
24	*Myrcianthes rhopaloides* (Kunth). Myrtaceae	PPN-my-001	Cold	Artisan	Stems	It is used in agriculture
25	*Paepalanthus ensifolius* Mart. Eriocaulaceae	PPN-el-002	Nerves	Water stored	Leaves	Oral
26	*Sigesbeckia mandoni* Schult. Asteraceae	PPN-as-051	Diarrhea in children of 1 to 6 months of age	Wormed	Leaves	Topic
27	*Macleania rupestris* (Kunth) A. C. Ericaceae	PPN-er-005	Reduces diarrhea and general malaise	Juice or food directly	Fruit	Oral
28	*Drimys granadensis* L. f. Winteraceae	PPN-wn-001	Sore teeth	Crushed	Bark of the plant	Topic
29	*Zea mays* L. Poaceae	PPN-po-012	Reduces diarrhea and general malaise	Infusion	Hair of Z. mays dry	Oral
30	*Tropaeolum tuberosum* Ruiz & Pav. Tropaeolaceae	PPN-tr-001	Prostate	Cooked	Tubers	Oral:
31	*Salvia scutellarioides* K. Lamiaceae	PPN-la-014	Infection of wounds	Cooked	Leaves	Wash the affected site
32	*Mentha spicata* Lamiaceae	PPN-la-027	Colic stomach and cold	Cooked	Leaves	Oral
33	*Myrica pubescens* Humb. & Bonpl. Myricaceae	PPN-mr-001	Fever or cold caused by cold air or strong winds (locally known as “mal aire”, i.e., “bad air” in Spanish)	It is used across clean.	Leaves	Topical and oral
34	*Clematis haenkeana* C. Pres. Ranunculaceae	PPN-ra-003	Sore teeth	Crushed	Buds	Topic
35	*Rubus urticifolius* Poir. Rosaceae	PPN-ro-005	Gangrene	Crushed	Buds and/flowers	Oral
36	*Gaultheria erecta* Vent. Eriaceae	PPN-er-008	Physical exhaustion	Eats	Fruit	Oral
37	*Bidens andicola* Kunth. Asteraceae	PPN-as-005	Diminish fall back into illness after recovery (locally known as “recaída”)	Crushed	Whole plant	Oral
38	*Juglans neotropica* Diles. Juglandaceae	PPN-ju-001	Postpartum bath	Cooked	Leaves	Bath
39	*Chenopodium ambrosioides* L. Chenopodiaceae	PPN-ch-001	Gallbladder stones	Cooked	Whole plant	Oral
40	*Viola dombeyana* DC. Violaceae	PPN-vi-004	Nerves	Infusion	Flowers	Oral
41	*Fuchsia hybrida* hort. ex Siebert & Voss. Onagraceae	PPN-on-005	Nerves	Infusion	Flowers	Oral
42	*Viola arguta* Willd. ex Roem. & Schult. Violaceae	PPN-vi-002	Nerves	Infusion	Flowers	Oral
43	*Siphocampylus scandens* (Kunth) G. Campanulaceae	PPN-cp-001	Nerves	Infusion	Flowers	Oral
44	*Petroselinum crispum* (Mill.) Apiaceae	PPN-ap-003	Nerves	Crushed	Whole plant	Oral
45	*Disterigma alaternoides* (Kunth) Ericaceae	PPN-er-006	Physical exhaustion	Eats	Fruit	Oral
46	*Poterium sanguisorba* L. Rosaceae	PPN-ro-008	Nerves	Crushed	Whole plant	Oral
47	*Clinopodium* sp. Lamiaceae	PPN-la-024	Menstrual related abdominal pain and cold	Infusion	Whole plant	Oral
48	*Myrteola phylicoides* (Benth.) Myrtaceae	PPN-my-006	Fever or cold caused by cold air or strong winds (locally known as “mal aire”, i.e., “bad air” in Spanish)	It is used directly	Leaves	Topic
49	*Clinopodium taxifolium* (Kunth) Lamiaceae	PPN-la-002	Fever or cold caused by cold air or strong winds (locally known as “mal aire”, i.e. “bad air” in Spanish)	It is used directly	Leaves	Topic
50	*Achyrocline hallii* Hieron. Asteraceae	PPN-as-058	Injuries	Place in the affected site	Leaves	Topic
51	*Fuchsia hypoleuca* I. M. Johnst. Onagraceae	PPN-on-009	Nerves	Infusion	Flowers	Oral
52	*Cleome longifolia* C. Pres. Capparaceae	PPN-ck-001	Rheumatism	Crushed	Leaves	Topic
53	*Cestrum sendtnerianum* Mart. Solanaceae	PPN-so-003	Fever, headache and relapse	Infusion	Leaves	Oral
54	*Cestrum* sp. Solanaceae	PPN-so-004	Fever, headache and relapse	Infusion	Leaves	Oral
55	*Bidens pilosa* L. Asteraceae	PPN-as-002	Diminish fall back into illness after recovery (locally known as “recaída”)	Crushed	Whole plant	Oral
56	*Pontederia* sp. Pontederiaceae	PPN-pk-001	Sore teeth	Chew	Leaves	Topic
57	*Macrocarpaea lenae *J. R. Grant Gentianaceae	PPN-gn-003	Fever or cold caused by cold air or strong winds (locally known as “mal aire”, i.e., “bad air” in Spanish)	Clean	Leaves	Topic
58	*Piper ecuadorense* Sodiro. Piperaceae	PPN-pi-007	Fever or cold caused by cold air or strong winds (locally known as “mal aire”, i.e. “bad air” in Spanish)	Clean.	Leaves	Topic
59	*Cyphomandra betacea* (Cav.) Solanaceae	PPN-so-014	Infection of the throat	Cooked	Fruit	Oral
60	*Carica pubescens* Lenné & C. Koch. Caricaceae	PPN-cc-003	Nerves and diarrhea	Cooked	Fruit	Oral
61	*Melissa officinalis* L. Lamiaceae	PPN-la-004	Nerves	Crushed	Whole plant	Oral
62	*Physalis peruviana* L. Solanaceae	PPN-so-013	Cholesterol	Juices	Fruit	Oral
63	*Gaiadendron punctatum* (Ruiz & Pav.) Loranthaceae	PPN-lo-001	Strong cough	Infusion	Flowers	Oral
64	*Otholobium mexicanum* (L. f.) J. W. Fabaceae	PPN-fa-005	Diarrhea	Infusion or cooking	Flowers	Oral
65	*Cavendishia bracteata* (Ruiz & Pav.) Ericaceae	PPN-er-003	Feed	Eats	Fruit	Oral
66	*Arracacia xanthorrhiza* Bancr. Apiaceae	PPN-ap-001	Elimination of the placenta in cattle	Cooked	Leaves	Oral
67	*Cucurbita maxima* Dúchense. Cucurbitaceae	PPN-cu-005	Diarrhea in children of 1 to 6 months of age	Warmed in the hands palms	Leaves	Topic

^*∗*^ Plants used in mythological cases.

**Table 5:** Ethnopharmacological report of medicinal plants used by visionary “Rikuyhampiyachak.”

Number	Scientific name	Herbarium voucher	Medicinal use	Preparation	Used part	Administration
1	*Cyclanthera pedata* (L.) Schrad. Cucurbitaceae	PPN-cu-004	Diminish fall back into illness after recovery (locally known as “recaída”)	Crushed	Flowers	Oral
2	*Scirpus* sp. Cyperaceae	PPN-cy-004	Child restless and confused, and postpartum bath	Cooked	Whole plant	Bath
3	*Alnus acuminata* Kunth. Betulaceae	PPN-be-001	Headache	Heated	Leaves	Topic
4	*Amaranthus cruentus* L. Amaranthaceae	PPN-am-002	Menstrual related abdominal pain	Cooked	Leaves	Oral
5	*Nasturtium officinale* R. Br. Brassicaceae	PPN-br-002	Headache	Crushed	Whole plant	Oral
6	*Borago officinalis *L. Boraginaceae	PPN-bo-001	Diminish fall back into illness after recovery (locally known as “recaída”) and cough	Infusion	Flowers	Oral
7	*Triumfetta althaeoides* Lam. Tiliaceae	PPN-ti-001	Inflammation of the liver and kidneys	Infusion	Leaves	Oral
8	*Salvia scutellarioides* Kunth. Lamiaceae	PPN-la-014	Water of air^*∗*^	Crushed	Flowers	Oral
9	*Ageratum conyzoides* L. Asteraceae	PPN-as-031	Gangrene and infection	Crushed	Whole plant	Oral
10	*Centaurium erythraea* Rafn. Gentianaceae	PPN-gn-001	Malaise of the body	Infusion	Flowers	Oral
11	*Sonchus oleraceus* L. Asteraceae	PPN-as-037	Malaise of the body	Infusion	Whole plant	Oral
12	*Lepidium* sp. Brassicaceae	PPN-br-008	Diminish fall back into illness after recovery (locally known as “recaída”)	Crushed	Whole plant	Oral
13	*Cotula australis* (Sieber ex Spreng.) Hook. f. Asteraceae	PPN-as-054	Diminish fall back into illness after recovery (locally known as “recaída”)	Crushed	Whole plant	Oral
14	*Lepidium chichicara* Desv. Brassicaceae	PPN-br-004	Diminish fall back into illness after recovery (locally known as “recaída”)	Crushed	Whole plant	Oral
15	*Tagetes terniflora* Kunth, Nov. Asteraceae	PPN-as-006	Fever or cold caused by cold air or strong winds (locally known as “mal aire”, i.e., “bad air” in Spanish)	It is used directly	Leaves	Topic
16	*Urtica urens* L. Urticaceae	PPN-ur-004	Intestinal Infection	Infusion	Whole plant	Oral
17	*Oxalis peduncularis* Kunth. Oxalidaceae	PPN-ox-002	Infection of the throat	Crushed	Whole plant	Topic
18	*Diplostephium oblanceolatum* S. F. Blake Asteraceae	PPN-as-045	Malaise of the body	Cooked	Leaves	Oral
19	*Cupressus lusitanica* Mill. Cupressaceae	PPN-cp-001	Control baldness	Macerate	Fruit	Topic
20	*Dianthus caryophyllus* Caryophyllaceae	PPN-cd-001	Stomach pain	Infusion	Flowers	Oral
21	*Brassica oleracea* L. Brassicaceae	PPN-br-007	Infection liver and kidneys	Crushed	Stems	Oral
22	*Equisetum bogotense* Kunth. Equisetaceae	PPN-eq-001	Inflammation of the liver	Cooked	Whole plant	Oral
23	*Peperomia peltigera* C. DC. Piperaceae	PPN-pi-010	Headache	Warm Fire	Fruit	Inhalation
24	*Peperomia galioides* Kunth. Piperaceae	PPN-pi-004	Water of air^*∗*^	Crushed	Whole plant	Oral
25	*Baccharis oblongifolia* (Ruiz & Pav.) Pers. Asteraceae	PPN-as-047	Child restless and confused, postpartum bath	Cooked	Branches	Bath
26	*Oreocallis grandiflora* (Lam.) R. Br. Proteaceae	PPN-pr-001	Inflammation of the liver	Infusion	Flowers	Oral
27	*Niphogeton dissecta* (Benth.) J. F. Macbr Apiaceae	PPN-ap-010	Cold	Cooked	Whole plant	Oral
28	*Apium leptophyllum *L. Apiaceae	PPN-ap-006	Cold	Cooked	Whole plant	Oral
29	*Adiantum poiretii* Wikstr. Pteriadaceae	PPN-pt-001	Cold	Cooked	Whole plant	Oral
30	*Iresine herbstii* Hook. Amaranthaceae	PPN-am-001	Infection: intestinal, liver and kidneys	Cooked	Whole plant	Oral
31	*Eucalyptus globulus* Labill. Myrtaceae	PPN-my-007	Fever or cold caused by cold air or strong winds (locally known as “mal aire”, i.e., “bad air” in Spanish)	Directly	Branches	Topic
32	*Epidendrum fimbriatum* Kunth. Orchidaceae	PPN-or-001	For internal tumors	Crushed	Flowers	Oral
33	*Rumex tolimensis* Wedd. Polygonaceae	PPN-pl-005	Dandruff	Crushed	Leaves	Topic
34	*Vicia faba* L. Fabaceae	PPN-fa-016	Headache	Boiled in the hands palms	Leaves	Topic
35	*Halenia weddelliana* Gilg. Gentianaceae	PPN-gn-002	It helps maintain milk production in cattle	Cooked	Whole plant	Topic
36	*Marchantia polymorpha *L. Amaranthaceae	PPN-am-008	Malaise of the body	Crushed	Whole plant	Oral
37	*Diplostephium* sp. Asteraceae	PPN-as-056	To bad energy^*∗*^	Cooked	Branches	Bath
38	*Tagetes erecta* L. Asteraceae	PPN-as-019	Water of air^*∗*^	Crushed	Flowers	Oral
39	*Myrica parvifolia* Benth. Myricaceae	PPN-mr-002	Fever or cold caused by cold air or strong winds (locally known as “mal aire”, i.e., “bad air” in Spanish)	Directly	Branches	Topic
40	*Gamochaeta americana* (Mill.) Wedd. Asteraceae	PPN-as-030	cold	Cooked	Whole plant	Oral
41	*Linum usitatissimum* L. Linaceae	PPN-li-001	Inflammation of liver and kidneys	Cooked	Fruits	Oral
42	*Alcea rosea* L. Malvaceae	PPN-ma-001	Inflammation of liver and kidneys	Infusion	Flowers	Oral
43	*Matricaria chamomilla* L. Asteraceae	PPN-as-016	Gastritis	Boiled	Whole plant	Oral
44	*Ambrosia artemisioides *Mill. Asteraceae	PPN-as-022	Fever or cold caused by cold air or strong winds (locally known as “mal aire”, i.e. “bad air” in Spanish)	Directly	Branches	Topic
45	*Piper aduncum* L. Piperaceae	PPN-pi-016	Infection of external wound	Cooked the leaves		Topic
46	*Diplostephium juniperinum* Cuatrec. Asteraceae	PPN-as-057	Child restless and confused, and postpartum bath	Cooked	Branches	Bath
47	*Eriocaulon microcephalum* Kunth, Erioculaceae	PPN-el-001	To luck good^*∗*^	Macerate	Whole plant	Inhalation
48	*Rubus urticifolius* Poir. Rosaceae	PPN-ro-005	Gangrene	Crushed	Flowers	Oral
49	*Bidens andicola* Kunth. Asteraceae	PPN-as-005	Diminish fall back into illness after recovery (locally known as “recaída”)	Crushed	Whole plant	Oral
50	*Pedicularis incurva* Benth. Scrophulariaceae	PPN-sc-004	Cold	Macerate	Branches	Oral
51	*Lepidium chichicara* Desv. Brassicaceae	PPN-br-004	Diminish fall back into illness after recovery (locally known as “recaída”)	Crushed	Whole plant	Oral
52	*Bejaria aestuans* Mutis ex L. Ericaceae	PPN-er-001	Menstrual related abdominal pain	Cooked	Flowers	Oral
53	*Bejaria subsessilis* Benth. Ericaceae	PPN-er-007	Nerves	Cooked	Flowers	Oral
54	*Fuchsia hybrida* hort. ex Siebert & Voss. Onagraceae	PPN-on-005	Nerves	Cooked	Flowers	Oral
55	*Poterium sanguisorba* L. Rosaceae	PPN-ro-008	Nerves	Crushed	Whole plant	Oral
56	*Pinus radiata* D. Pinaceae	PPN-pc-001	Asthma	Cooked	Fruit	Oral
57	*Clinopodium* sp. Lamiaceae	PPN-la-024	Cold	Cooked	Whole plant	Oral
58	*Minthostachys mollis* (Kunth) Grises. Lamiaceae	PPN-la-009	Fever or cold caused by cold air or strong winds (locally known as “mal aire”, i.e., “bad air” in Spanish)	Directly	Branches	Topic
59	*Chrysanthemum* sp. Asteraceae	PPN-as-055	Fever or cold caused by cold air or strong winds (locally known as “mal aire”, i.e. “bad air” in Spanish)	Crushed	Leaves	Oral
60	*Ceroxylon parvifrons* (Engel) H. Wendl. Aricaceae	PPN-ak-001	The aerial part is used as incense^*∗*^	Burns	Leaves	
61	*Rosmarinus officinalis* L. Lamiaceae	PPN-la-010	Fever or cold caused by cold air or strong winds (locally known as “mal aire”, i.e., “bad air” in Spanish)	Crushed	Branches	Oral
62	*Rosa centifolia* L. Rosaceae	PPN-ro-001	Nerves	Crushed	Flowers	Oral
63	*Ruta graveolens* L. Rutaceae	PPN-rt-001	Headache, bad air^*∗*^	Crushed	Branches	Oral
64	*Solanum juglandifolium* Dunal, Solan. Solanaceae	PPN-so-016	Air water^*∗*^	Crushed	Flowers	Oral
65	*Echinopsis pachanoi* (Britton & Rose) Cactaceae	PPN-cb-001	Sorcery^*∗*^	Cooked	Stems	Oral
66	*Tanacetum parthenium *(L.) Sch. Bip. Asteraceae	PPN-as-031	Fright in children	Cooked	Whole plant	Oral
67	*Brachyotum confertum* (Bonpl.) Triana. Melastomataceae	PPN-me-004	Allergies	Crushed and cook	Branches	Topic
68	*Cestrum sendtnerianum* C. Martius. Solanaceae	PPN-so-003	Fever, headache and relapse	Infusion	Leaves and flowers	Oral
69	*Baccharis obtusifolia* Kunth. Asteraceae	PPN-as-014	Fever or cold caused by cold air or strong winds (locally known as “mal aire”, i.e., “bad air” in Spanish)	Directly	Branches	Topic
70	*Baccharis *sp. Asteraceae	PPN-as-015	Fever or cold caused by cold air or strong winds (locally known as “mal aire”, i.e., “bad air” in Spanish)	Directly	Branches	Topic
71	*Lepechinia paniculata* (Kunth). Lamiaceae	PPN-la-011	Fever or cold caused by cold air or strong winds (locally known as “mal aire”, i.e., “bad air” in Spanish)	Directly	Branches	Topic
72	*Bidens pilosa *L. Asteraceae	PPN-as-002	Diminish fall back into illness after recovery (locally known as “recaída”)	Crushed	Flowers	Oral
73	*Tagetes erecta* L. Asteraceae	PPN-as-019	Air water^*∗*^	Crushed	Flowers	Oral
74	*Baccharis genistelloides* (Lam.) Pers. Asteraceae	PPN-as-013	Diabetes and cholesterol	Cooked	Branches	Oral
75	*Piper barbatum* Kunth. Piperaceae	PPN-pi-005	Fever or cold caused by cold air or strong winds (locally known as “mal aire”, i.e. “bad air” in Spanish)	Directly	Branches	Topic
76	*Baccharis genistelloides* (Lam.) Pers. Asteraceae	PPN-as-013	Diabetes and cholesterol	Cooked	Branches	Oral
77	*Iresine herbstii* Hook. Amaranthaceae	PPN-am-001	Flu and bad air^*∗*^	Cooked	Whole plant	Oral
78	*Clinopodium nubigenum* (Kunth). Lamiaceae	PPN-la-018	Cold	Infusion	Whole plant	Oral
79	*Melissa officinalis* L. Lamiaceae	PPN-la-004	Nerves	Crushed	Branches	Oral
80	*Huperzia* sp. Lycopodiaceae	PPN-lc-007	Child restless and confused, and postpartum bath	Cooked	Branches	Bath
81	*Huperzia tetragona* (Hook. & Grev.) Lycopodiaceae	PPN-lc-004	Sorcery^*∗*^	Macerate	Whole plant	Oral
82	*Solanum oblongifolium* Dunal, Solan. Solanaceae	PPN-so-014	Fever or cold caused by cold air or strong winds (locally known as “mal aire”, i.e. “bad air” in Spanish)	Directly	Branches	Topic
83	*Oritrophium peruvianum* (Lam.) Asteraceae	PPN-as-046	Inflammation of the liver and kidneys	Cooked	Whole plant	Oral
84	*Oritrophium peruvianum* (Lam.) Asteraceae	PPN-as-046	Inflammation of the liver and kidneys	Cooked	Whole plant	Oral
85	*Oritrophium peruvianum* (Lam.) Asteraceae	PPN-as-046	Inflammation of the liver and kidneys	Cooked	Whole plant	Oral
86	*Loricaria thuyoides* (Lam.) Sch. Asteraceae	PPN-as-044	Child restless and confused, bath and good energy^*∗*^	Cooked	Branches	Bath
87	*Valeriana microphylla* Kunth. Valerianaceae	PPN-va-001	Nerves	Cooked	Roots	Oral
88	*Verbena litoralis *Kunth. Verbenaceae	PPN-ve-001	Plague and headache	Crushed	Flowers	Oral
89	*Huperzia* sp. Lycopodiaceae	PPN-lc-007	Amulet for evil eye and sorcery^*∗*^	Macerate	Whole plant	Oral
90	*Huperzia sellifolia* B. Øllg. Lycopodiaceae	PPN-lc-002	Amulet for evil eye and sorcery^*∗*^	Macerate	Whole plant	Oral
91	*Lycopodium weberbaueri* (Nessel). Lycopodiaceae	PPN-lc-005	Amulet for evil eye and sorcery^*∗*^	Macerate	Whole plant	Oral
92	*Huperzia austroecuadorica* B. Øllg. Lycopodiaceae	PPN-lc-006	Amulet for evil eye and sorcery^*∗*^	Macerate	Whole plant	Oral
93	*Brugmansia X candida* Pers. Solanaceae	PPN-so-015	Fever or cold caused by cold air or strong winds (locally known as “mal aire”, i.e., “bad air” in Spanish)	Directly	Directly	Topic
94	*Daucus carota* L. Apiaceae	PPN-um-001	Gastritis	Juice	Drops	Oral

^*∗*^Plants used in mythological cases.

**Table 6:** Ethnopharmacological reports of medicinal species used by a bone healer “Kakuyhampiyachak.”

Number	Scientific name	Herbarium voucher	Medicinal use	Preparation	Used part	Administration
1	*Persea americana* Mill. Lauraceae	PPN-lu-001	Coups and hematomas	Scraped	Seed	Oral
2	*Alnus acuminata* Kunth. Betulaceae	PPN-be-001	Rupture of bones, sprains and dislocations	Crushed	Buds	Topic
3	*Urtica urens* L. Urticaceae	PPN-ur-004	Blows	Crushed	Whole plant	Topic
4	*Oreocallis grandiflora* (Lam.) R. Br. Proteaceae	PPN-pr-001	Twists and blows	Crushed	Fruit	Topic
5	*Solanum americanum* Mill. Solanaceae	PPN-so-007	Blows internal	Cooked	Leaves	Oral
6	*Chenopodium album* L. Chenopodiaceae	PPN-ch-002	Blows, dislocation, sprains	Crushed	Branches or buds	Topic
7	*Agave americana* L. Amaryllidaceae	PPN-ar-002	Bone fracture and dislocation	Gets small slats	Stems	Topic
8	*Cucurbita ficifolia* Bouché, Verh. Amaryllidaceae	PPN-cu-001	Blows	It uses the buds pounded and mixed with natural sweetener (panela)	Whole plant	Topic
9	*Phoradendron parietarioides* Trel. Viscaceae	PPN-vs-002	Bone fractures and dislocated	Crushed	Whole plant	Topic
10	*Dendrophthora fastigiata* Kuijt. Viscaceae	PPN-vs-001	Bone fractures and dislocated	Crushed	Whole plant	Topic
11	*Carica pubescens* Lenné & C. Koch. Caricaceae	PPN-cc-003	Dislocation	Heat	Leaves	Topic
12	*Solanum oblongifolium* Dunal, Solan. Solanaceae	PPN-so-014	Dislocation	Heat	Leaves	Topic

**Table 7:** Ethnopharmacological reports of medicinal species used by a midwife “Wachackhampiyachak.”

Number	Scientific name	Herbarium voucher	Medicinal use	Preparation	Used part	Administration
1	*Oxalis corniculata* L. Oxalidaceae	PPN-ox-001	Scurvy "scorbutic tongue"	To crush	Whole plant	Topic
2	*Impatiens* sp. Balsaminaceae	PPN-ba-001	Postpartum relapse	Infusion	Flowers	Oral
3	*Impatiens balsamina* L. Balsaminaceae	PPN-ba-001	Postpartum relapse	Infusion	Flowers	Oral
4	*Begonia* sp. Begoniaceae	PPN-ba-001	Postpartum relapse	Infusion	Flowers	Oral
5	*Impatiens balsamina* L. Balsaminaceae	PPN-ba-001	Postpartum relapse	Infusion	Flowers	Oral
6	*Nasturtium officinalis* R. Br. Brassicaceae	PPN-br-002	Malaise of the body and flu	Crushed	Whole plant	Oral
7	Borago officinalis L. Boraginaceae	PPN-bo-001	Postpartum relapse and cough	Infusion	Flowers	Oral
8	*Tradescantia zebrina* Heynh. Commelinaceae	PPN-co-004	Postpartum relapse	Crush	Whole plant	Oral
9	*Callisia repens* (Jacq.) L. Commelinaceae	PPN-co-001	Postpartum relapse	Crush	Whole plant	Oral
10	*Ageratum conyzoides* L. Asteraceae	PPN-as-037	Gangrene and infections after birth	Crushed	Whole plant	Oral
11	*Geranium diffusum* Kunth. Geraniaceae	PPN-ge-010	Gangrene and infections after birth	Crushed	Whole plant	Oral
12	*Lepidium chichicara* Desv. Brassicaceae	PPN-br-004	Fever or cold caused by cold air or strong winds (locally known as “mal aire”, i.e., “bad air” in Spanish)	Crushed	Whole plant	Oral
13	*Dianthus caryophyllus* L. Caryophyllaceae	PPN-cd-001	Stomach ache	Infusion	Flowers	Oral
14	*Brassica oleracea* L. Brassicaceae	PPN-br-007	Postpartum infection	Crushed	Stem	Oral
15	Peperomia peltigera C. DC. Piperaceae	PPN-pi-010	Nerves and headache	Infusion	Leaves	Oral
16	*Mesembryanthemum elegans* L. Aizoaceae	PPN-az-002	Nerves and headache	Infusion	Leaves	Oral
17	Peperomia Inaequalifolia Ruiz & Pav. Piperaceae	PPN-pi-009	Fright children	Cooked	Leaves	Bath
18	*Taraxacum officinale* F. H. Wigg. Asteraceae	PPN-as-020	Gastritis, ulcer and cleanse	Infusion	Whole plant	Oral
19	*Iresine herbstii* Hook. Asteraceae	PPN-am-001	Infections of uteri, vagina, liver and kidney	Crushed	Stem and leaves	Oral
20	*Pelargonium* sp. Geraniaceae	PPN-ge-008	Cold and nervous during childbirth and postpartum	Infusion	Leaves	Oral
21	*Pelargonium graveolens* L'Hér. Geraniaceae	PPN-ge-004	Cold and nervous during childbirth and postpartum	Infusion	Leaves	Oral
22	*Pelargonium zonale* (L.) L'Hér. Geraniaceae	PPN-ge-005	Infections, vaginal, before childbirth and postpartum	Crushed	Flowers	Topic
23	*Foeniculum vulgare* Mill. Apiaceae	PPN-ap-004	Increase maternal milk, indigestion, colic menstrual	Infusion	Whole plant	Oral
24	*Myrica parvifolia* Benth. Myricaceae	PPN-mr-002	Bad air^*∗*^, colic stomach, to treat faint during childbirth	Chew	Buds	Oral
25	*Linum usitatissimum *L. Linaceae	PPN-li-001	Inflammation of liver and kidney	Cooked	Fruit	Oral
26	*Plantago major* L. Plantaginaceae	PPN-pn-001	Inflammation of liver and kidney	Cooked	Whole plant	Oral
27	*Anredera ramosa* (Moq.) Eliasson. Basellaceae	PPN-bs-001	Bath the children, fever, headache	Crushed and to scrub in hot water	Whole plant	Bath
28	*Lavatera arborea* L. Malvaceae	PPN-ma-009	Inflammation of liver and kidney	Infusion	Flowers	Oral
29	*Pelargonium odoratissimum *L. Geraniaceae.	PPN-ge-001	Cold during childbirth	Cooked or infusion	Branches	Oral
30	*Mentha pulegium* L. Lamiaceae	PPN-la-015	Colic stomach, indigestion and cold	Infusion	Branches	Oral
31	*Mentha piperita* subsp. Lamiaceae	PPN-la-006	Colic stomach, indigestion and cold	Infusion	Branches	Oral
32	*Bidens andicola* Kunth. Asteraceae	PPN-as-005	Postpartum relapse	Crushed	Whole plant	Oral
33	*Thymus vulgaris* L. Lamiaceae	PPN-la-022	Indigestion	Cooked	Branches	Oral
34	*Ageratina dendroides* (Spreng) R. M. Asteraceae	PPN-as-053	Coups and extraction of pus	Put the place affected	Buds	Topic
35	Fuchsia hybrida Hort. Onagraceae	PPN-on-005	Nerves during childbirth and postpartum	Infusion	Flowers	Oral
36	*Viola tricolor *L. var 1. Violaceae	PPN-vi-003	Nerves	Infusion	Flowers	Oral
37	*Viola tricolor* L. var 2. Violaceae	PPN-vi-003	Nerves	Infusion	Flowers	Oral
38	*Clinopodium* sp. Lamiaceae	PPN-la-024	Colic menstrual and allergy	Infusion	Whole plant	Oral/to rub
39	*Ruta graveolens* L. Rutaceae	PPN-rt-001	Bath, fain during childbirth	Crushed	Flowers	Oral
40	*Tanacetum parthenium *(L.) Sch. Bip. Asteraceae	PPN-as-031	Children fright	Cooked	Whole plant	Bath
41	*Cestrum sendtnerianum* C. Martius. Solanaceae	PPN-so-003	Postpartum relapse	Infusion	Flowers	Bath
42	*Baccharis obtusifolia* Kunth. Asteraceae	PPN-as-014	Cold during childbirth.	Burn the dry	Leaves	Topic
43	*Salvia leucocephala* Kunth. Lamiaceae	PPN-la-025	Postpartum bath	Cooked	Whole plant	Bath
44	*Iresine herbstii* Hook. var 1. Amaranthaceae	PPN-am-001	Flu, fever or cold caused by cold air or strong winds (locally known as “mal aire”, i.e., “bad air” in Spanish)	Infusion	Whole plant	Oral
45	*Iresine herbstii* Hook. var 2. Amaranthaceae	PPN-am-001	Flu, fever or cold caused by cold air or strong winds (locally known as “mal aire”, i.e., “bad air” in Spanish)	Infusion	Whole plant	Oral
46	*Iresine herbstii* Hook var 3. Amaranthaceae	PPN-am-001	Flu, fever or cold caused by cold air or strong winds (locally known as “mal aire”, i.e., “bad air” in Spanish)	Infusion	Whole plant	Oral
47	*Melissa officinalis* L. Lamiaceae	PPN-la-004	Nerves	Crushed	Whole plant	Oral
48	*Verbena litoralis *Kunth. Verbenaceae	PPN-ve-001	Malaise of the body, infection of the throat, and flu	Cooked	Whole plant	Oral
49	*Viola odorata* L. Violaceae	PPN-vi-001	Cough	Infusion	Flowers	Oral

^*∗*^Plants used in mythological cases.
